# Perceived social support, caregiver capacity, and socioeconomic determinants mediating pathways to family resilience in Chinese stroke survivors: a cross-sectional study

**DOI:** 10.1186/s12912-025-03826-y

**Published:** 2025-09-26

**Authors:** Yiqing Zhang, Jingjing Ma, Lijun Chen, Huiying Chen, Yamei Xu, Hong Xu, Lei Gu, Lu Shi

**Affiliations:** 1 Department of Nursing, Ningbo Medical Center LiHuili Hospital, Ningbo, 315000 China; 2 Department of Medicine, Ningbo Medical Center LiHuili Hospital, Ningbo, 315000 China; 3 Department of Medical, Ningbo Medical Center LiHuili Hospital, Ningbo, 315000 China

**Keywords:** Family resilience, Stroke survivors, Caregivers, Perceived social support, Caregiver capacity, Socioeconomic determinants, Cross-Sectional study, Mediating role

## Abstract

**Background:**

Family resilience theory posits that families can cultivate adaptive capacities to thrive amid adversity. However, evidence on family resilience mechanisms among Chinese stroke survivors and their caregivers remains limited.

**Objectives:**

To investigate the direct and indirect pathways linking perceived social support, caregiver capacity, and family resilience in stroke-affected families and to identify socioeconomic determinants shaping resilience outcomes.

**Design:**

This is a cross-sectional study.

**Methods:**

A convenience sample of 513 stroke survivor-caregiver dyads was recruited from two hospitals in Ningbo, China: a large public hospital and a private rehabilitation hospital. Quantitative data were collected using a structured questionnaire with respondent-stratified data sources. Primary family caregivers self-reported their general demographics, as well as their responses to the Perceived Social Support Scale (PSSS), Family Caregiver Task Inventory (FCTI), and Family Resilience Assessment Scale (FRAS-C). Concurrently, the demographics, clinical characteristics, and Activities of Daily Living (ADLs) of stroke survivors were assessed. Statistical analyses encompassed descriptive statistics, Pearson correlation coefficients, multivariate regression models, and mediation analysis to examine relationships between variables.

**Results:**

Family resilience scores (mean = 86.70 ± 19.00) showed significant positive correlations with perceived social support (PSS) (*r* = 0.523, *p* < 0.01) and positive correlations with caregiver capacity (FCTI, *r* = 0.522, *p* < 0.01). Mediation analysis revealed that caregiver capacity partially mediated the relationship between PSS and family resilience (indirect effect: 26.68%). Socioeconomic determinants played a critical role: higher education (β = 8.50, *p* = 0.008), medical insurance coverage (β = -6.47, *p* = 0.009), and mild stroke survivors dependence (ADL;β = 9.40, *p* < 0.001) enhanced resilience, while higher pre-illness income of stroke survivors (β = -8.31, *p* = 0.002) and poor caregiver sleep quality (β = -6.20, *p* < 0.05) reduced it.

**Conclusion:**

This study provides novel evidence on the psychosocial mechanisms underlying family resilience in Chinese stroke populations. First, we identify caregiver capacity as a critical mediator between social support and resilience, elucidating a previously underexplored pathway. Second, we reveal the paradoxical association between higher pre-stroke income and reduced resilience, challenging conventional socioeconomic assumptions in resilience research. Third, we establish caregiver sleep quality as a modifiable determinant of family resilience. The study suggests that interventions should simultaneously strengthen social support networks and caregiver skills, while addressing issues related to sleep quality and financial concerns.

**Clinical trial number:**

Not applicable.

**Supplementary Information:**

The online version contains supplementary material available at 10.1186/s12912-025-03826-y.

## What is already known

- Family resilience plays a crucial role in post-stroke recovery, enabling families to cope with and adapt to the challenges of adversity.
- Perceived social support and caregiver capacity are established psychosocial factors that contribute to family resilience.
- Existing studies often focus on stroke survivors-centered outcomes, with limited integration of socioeconomic and psychosocial factors in family resilience frameworks, particularly in non-Western contexts like China.

**Supplementary Information:**

The online version contains supplementary material available at 10.1186/s12912-025-03826-y.

## What this paper adds

- Mediation pathway: Identifies caregiver capacity as a partial mediator (26.68% indirect effect) between social support and family resilience, offering a novel psychosocial pathway.
- Socioeconomic determinants: Highlights unique socioeconomic factors (e.g., higher education enhancing resilience, medical insurance reducing financial strain, and higher pre-illness income paradoxically lowering resilience) in the Chinese context.
- This study advances the Family Resilience Framework by integrating socioeconomic determinants and caregiver dynamics, providing a holistic model for intervention design in similar contexts.

## Introduction

Stroke is a leading cause of long-term disability worldwide. Globally, over 12.2 million new stroke cases occur annually, with the total number of stroke patients reaching 101 million [1]. China bears a significant portion of this burden, reporting 3.94 million new stroke cases each year, which represents nearly one-third of the global total [2]. Post-stroke complications, including movement disorders and speech impairments, are common [3]. These complications often create long-term care demands and disrupt family structure and function [4]. Studies show that effective family adjustment can facilitate patient recovery and enhance family life quality [5]. Family resilience refers to a family’s ability to adapt positively and thrive in the face of major adversities. It enables families to maintain stability through successful adjustment [6]. Strengthening family resilience is therefore essential for sustaining long-term care capabilities and restoring family functioning after stroke.

Numerous studies have identified key factors influencing family resilience [7], including economic pressure, role renegotiation, and emotional distress. These factors can threaten family stability and hinder recovery trajectories. However, significant gaps remain in understanding the specific mechanisms of family resilience development [8, 9], particularly within China’s unique cultural context. In China, families serve as the primary care unit, with family resilience influenced not only by external resource availability but also by members’ subjective perceptions and interaction patterns.

Perceived Social Support (PSS) refers to an individual’s subjective evaluation of the support available from family, friends, and community networks [10]. Existing research demonstrates that PSS serves as a critical protective factor for family resilience in the context of stroke [11, 12]. For caregivers, strong perceived support can reduce emotional exhaustion while sustaining motivation and well-being. Specifically, it mitigates caregiver stress and burden, thereby strengthening family resilience.

Caregiver capacity refers to a caregiver’s ability to effectively address the health needs of stroke survivors while fulfilling their physical, emotional, and social requirements [13]. This capacity includes the caregiver’s knowledge, skills, available resources, and support systems. Research demonstrates that caregiver capacity has a significant impact on stroke recovery outcomes [14, 15]. Effective caregiving is particularly crucial for meeting the complex rehabilitation needs of survivors. In addition, socioeconomic factors such as income level, education, and healthcare access significantly affect how families adapt to stroke-related challenges [16–18]. These fundamental elements work together to determine a family’s capacity for caregiving and their overall resilience.

Current research acknowledges the significance of PSS and caregiver capacity in promoting family resilience, yet several gaps remain. First, existing measurement tools fail to distinguish between perceived support and objective support clearly. For instance, in the study on the correlation between social support and family resilience of stroke caregivers [19], the social support rating scale used included both objective support items and subjective experience items, making it challenging to capture the core of PSS. Second, the mechanisms linking PSS to family resilience remain unclear. While studies suggest that it helps by improving caregiver confidence, reducing loneliness, or promoting adaptive coping [20, 21], most focus only on single dimensions, such as emotional support. Caregiver capacity is multidimensional [22], involving health literacy, practical skills, resource management, and role coordination. This broader perspective is often missing. Especially in the context of Chinese culture, traditional family concepts not only maintain the continuity of care but may also intensify emotional depression, which makes the psychological support role of PSS particularly prominent. Based on this, we propose that caregiver ability may be a key mediating variable connecting PSS and family resilience, but there is currently a lack of systematic theoretical construction and empirical testing.

Walsh’s Family Resilience Framework [23] identifies three core mechanisms that help families of stroke survivors enhance their adaptability. First, the belief system serves as the cognitive foundation. It influences how caregivers perceive challenges. When family members view stroke as a manageable challenge rather than an insurmountable obstacle, their psychological adaptability significantly improves [24]. Second, the organizational pattern involves dynamic family restructuring. Studies indicate that redistributing caregiving responsibilities effectively supports patient recovery [25]. Third, communication processes play a vital role. Open emotional expression reduces caregiver depression risk, and collaborative problem-solving also mediates between socioeconomic status and psychological resilience [26]. These three interrelated mechanisms constitute the framework that supports family adaptation.

The Social-Ecological Model (SEM) [27] provides a structured framework for understanding how environmental systems influence family resilience. At the micro level, caregiver capacity directly corresponds to the organizational patterns within the Family Resilience Framework. The capacity, along with caregiver-patient interactions, significantly affects the adaptation outcome [28]. At the meso level, PSS constitutes an important environmental support, including elements such as community networks and the accessibility of medical services. Studies have shown that effective interaction among doctors, patients, and their families can significantly improve the quality of rehabilitation [26]. Community support groups also effectively reduce family stress [29]. At the macro level, social and economic policies, as well as cultural norms, have shaped fundamental constraints. For example, Confucian filial piety norms may inadvertently increase caregiver burden through role expectations [30]. This multilevel analysis reveals how structural factors interact with family processes to shape resilience outcomes.

In China, where resources vary by region, families often rely on community networks to compensate for the lack of formal support. This reality underscores how family resilience emerges not merely from internal coping strategies but is fundamentally shaped by external environmental conditions. This study integrates Walsh’s Family Resilience Framework with Bronfenbrenner’s Social-Ecological Model to build a theoretical foundation.

Using a cross-sectional design, this study investigated the family resilience of stroke survivors and their caregivers. It examined the roles of social support, caregiving capacity, and socioeconomic factors in shaping family resilience, aiming to understand how these dynamic factors influence family resilience. Research hypothesis: (H1) Family resilience in stroke survivors shows associations with both caregiving capacity and PSS. (H2) Caregiving capacity may mediate the relationship between PSS and family resilience. The findings from this study will provide a theoretical basis for formulating intervention plans aimed at enhancing the family resilience of stroke survivors.

## Methods

### Participants

This study was conducted at two hospitals in Ningbo, China. The first is a large public hospital, which serves as the region’s primary tertiary referral center. It caters to a diverse patient population, spanning both urban and rural areas, and addresses a broad spectrum of medical needs, ranging from acute to chronic conditions. The second is a private rehabilitation hospital. It specializes in post-acute care and long-term functional recovery. These two settings, together, represent important components of the healthcare system. They cover care from initial treatment to ongoing rehabilitation. We recruited stroke survivors and their primary family caregivers from both hospitals. Participants were recruited using a convenience sampling method. The recruitment period is from October 18, 2024, to February 18, 2025. The final sample included 513 hospitalized stroke survivors and their paired primary family caregivers. The primary caregiver is defined as an unpaid family member (spouse, adult children, parents, or other relatives) designated by a stroke survivor as the primary source of daily support during hospitalization [31].

Criteria for stroke survivors: Inclusion: (1) Diagnosis confirmed by “Diagnosis of Major Types of Cerebrovascular Diseases” (2019); (2) Age ≥ 18 years; (3) Clinically stable, defined as: no ICU transfer, emergency interventions (e.g., reintubation, vasopressors), or neurological deterioration within 72 h prior to enrollment; (4) Voluntary consent. Exclusion: (1) Severe systemic diseases (e.g., metastatic cancer) or altered consciousness; (2) Withdrawal or refusal during the study.

Criteria for primary caregivers: Inclusion: (1) Unpaid family member (spouse, adult child, parent) designated by the stroke survivors as the primary hospital companion; (2) Assisted with daily care tasks (e.g., feeding, positioning) as delegated and supervised by nursing staff; (3) Age ≥ 18 years; (4) Voluntary consent. Exclusion: (1) Hired professionals or non-primary caregivers (e.g., occasional visitors); (2) Severe physical/mental health conditions impairing caregiving ability.

### Sample size

Our sample size determination for testing the mediation model followed rigorous methodological standards for causal pathway analysis. Based on conservative estimates of small-to-medium effect sizes (a = 0.26, b = 0.39; ab = 0.10) from meta-analytic data [32] and accounting for the joint significance requirement of mediation analysis, we conducted comprehensive power calculations incorporating both analytical approaches (Sobel test approximation yielding *N* = 287) and empirical validation through Monte Carlo simulations (10,000 iterations in Mplus). These analyses indicated that our final sample of *N* = 513 provides more than 90% power to detect the hypothesized indirect effect (power = 0.94 with percentile confidence interval), while maintaining robustness against potential 15% effect size underestimation and 10% missing data.

### Measures

This study employed a structured questionnaire with respondent-stratified data sources: primary family caregivers self-reported general demographics, Perceived Social Support Scale (PSSS), Family Caregiver Task Inventory (FCTI), and Family Resilience Assessment Scale (FRAS-C); concurrently, survivor demographics, clinical characteristics (from medical records/structured interviews), and Activities of Daily Living (ADL). These traits are significant for families facing the challenges of stroke. The questionnaire effectively assesses and explains these complex psychological mechanisms. Collectors must undergo training in research objectives and ethics, questionnaire content, communication skills, and handling participant inquiries Supplementary File 1.

Demographic characteristics, including gender, age, education level, and income.

The ADL assessment evaluates a person’s independence in daily tasks, such as hygiene, dressing, and eating, with scores ranging from 0 to 100 [33]. Higher scores indicate greater independence: 61–99 scores reflect mild dependence, where some help may be needed; 41–60 scores indicate moderate dependence, requiring significant assistance; ≤40 scores indicate severe dependence, where the individual cannot perform most activities without full assistance.

The FRAS-C has 32 items, divided into three subscales: family communication and problem-solving, utilizing social resources, and maintaining a positive attitude. Each item is rated on a four-point Likert scale (1 = strongly disagree to 4 = strongly agree), with total scores ranging from 32 to 128; higher scores indicate greater family resilience. The scale’s Cronbach’s alpha is 0.88–0.96, and the validity coefficients range from 0.75 to 0.86 [34]. In this study, the Cronbach’s alpha was 0.93.

The PSSS measure assesses an individual’s perception of social support from their network, including family, friends, and significant others. It consists of 12 items rated on a scale from 1 (very strongly disagree) to 7 (very strongly agree) [35]. The PSSS has demonstrated high reliability in previous studies, with a Cronbach’s alpha coefficient of 0.914 [36]. In this study, the Cronbach’s alpha for the PSSS was 0.923.

The FCTI assesses caregiver capacity, including adapting to the caregiver role, responding flexibly to provide care, managing personal emotions, assessing family and community resources, and adjusting life to meet caregiving needs. Each dimension comprises five items, totaling 25 items. Responses are recorded on a five-point Likert scale, ranging from “Not difficult at all” (one point) to “Extremely difficult” (five points). To facilitate data interpretation while maintaining analytical validity, the scoring was reversed in this study, with “Extremely difficult” assigned five points and “Not difficult at all” assigned one point. Consequently, higher scores indicate stronger family caregiving ability. In previous studies, Cronbach’s alpha coefficient was 0.93 [37]. In this study, the Cronbach’s alpha was 0.93.

### Outcome variable definition

Our analysis examined the relationships among caregiver capacity, family resilience, and PSS. PSS was treated as the independent variable, caregiver capacity as the mediator, and family resilience as the primary outcome of interest. The analysis included sociodemographic characteristics and ADL scores as covariates to identify factors influencing family resilience. In the mediation analysis, these covariates were considered to control for potential confounding factors.

### Statistical analysis

Data processing and analysis were performed using R version 4.4.0 (2024-04-24), along with Zstats 1.0 (www.zstats.net), an R-based statistical tool with rigorously validated algorithms. Mediation analysis was performed using Jamovi version 2.3.0, which includes a mediation module. The study employed descriptive analysis, independent sample t-tests, one-way analysis of variance (ANOVA), Pearson correlation analysis, and multiple stepwise regression analysis. The mediation effect was tested using the mediation module in Jamovi version 2.3.0. All statistical tests were two-tailed, with a significance level set at *p* < 0.05. (1) Means ± standard deviations, frequencies, and composition ratios were used to describe the general characteristics of family resilience among stroke survivors and the scores of four variables. (2) Independent sample t-tests or one-way ANOVA were used to analyze differences in family resilience scores among stroke survivors based on demographic characteristics. (3) Pearson correlation analysis examined the relationships between PSSS, family resilience, and caregiver capacity. (4) Multiple stepwise regression analysis was conducted to identify factors influencing family resilience among stroke survivors. (5) A mediation model was constructed using Jamovi version 2.3.0 and its mediation module to verify the mediating effect of caregiver capacity on the relationship between PSSS and family resilience.

## Results

### The demographic and clinical characteristics of study participants

A total of 600 questionnaires were issued, and 530 were recovered. Seventeen questionnaires were excluded because the data was missing more significantly than 20%. The final sample included 513 survivor-caregiver pairs, with a participation rate of 85.5%. Of the 513 valid questionnaires, the participants comprised stroke survivors and their primary caregivers.

Table 1 presents the demographic and clinical characteristics of 513 stroke survivors, along with the associations between these characteristics and family resilience. Factors such as gender, age, education level, time since diagnosis, employment status, type of medical insurance, ADL, and income level were analyzed.

**Table 1:** Demographic and clinical characteristics of stroke survivors with family resilience

Variable	*n* (%)	FRAS, Mean ± SD	*P*	β (95%CI)
**Gender**				
Male	268 (52.24)	87.62 ± 18.55		0.00 (Reference)
Female	245 (47.76)	85.69 ± 19.47	0.252	-1.93 (-5.22 ∼ 1.37)
**Age(years)**				
18–40	48 (9.36)	84.35 ± 17.06		0.00 (Reference)
41–60	180 (35.09)	86.38 ± 19.73	0.512	2.03 (-4.03 ∼ 8.09)
61–80	198 (38.60)	87.70 ± 18.01	0.275	3.35 (-2.65 ∼ 9.35)
> 80	87 (16.96)	86.37 ± 20.78	0.557	2.01 (-4.69 ∼ 8.72)
**Education**				
Junior High School	360 (70.18)	85.81 ± 18.35		0.00 (Reference)
High School	116 (22.61)	87.20 ± 21.39	0.493	1.39 (-2.57 ∼ 5.35)
College and Above	37 (7.21)	93.78 ± 15.96	0.015	7.97 (1.57 ∼ 14.38)
**Time of diagnosis**				
< 1 month	220 (42.88)	89.55 ± 17.15		0.00 (Reference)
1–2 months	135 (26.32)	83.67 ± 19.87	0.005	-5.87 (-9.91 ∼ -1.83)
> 2 months	158 (30.80)	85.32 ± 20.23	0.032	-4.22 (-8.08 ∼ -0.37)
**Employ**				
Employed	134 (26.12)	87.64 ± 18.43		0.00 (Reference)
Resigned	80 (15.59)	86.49 ± 21.76	0.668	-1.15 (-6.42 ∼ 4.12)
Retired	299 (58.28)	86.33 ± 18.52	0.509	-1.31 (-5.19 ∼ 2.57)
**Medical**				
Rural medical insurance	144 (28.07)	89.11 ± 17.60		0.00 (Reference)
Urban medical insurance	229 (44.64)	88.22 ± 19.33	0.654	-0.89 (-4.80 ∼ 3.01)
Commercial insurance	47 (9.16)	86.15 ± 17.87	0.347	-2.96 (-9.13 ∼ 3.21)
Self-financing	93 (18.13)	79.51 ± 19.36	< 0.001	-9.61 (-14.49 ∼ -4.72)
**ADL**				
Moderate dependence	321 (62.57)	86.72 ± 17.15		0.00 (Reference)
Severe dependence	123 (23.98)	80.73 ± 20.50	0.002	-5.99 (-9.82 ∼ -2.16)
Mild dependence	69 (13.45)	97.23 ± 20.10	< 0.001	10.51 (5.72 ∼ 15.30)
**Monthly income before illness (in RMB)**				
< 3000 yuan	194 (37.82)	91.86 ± 14.88		0.00 (Reference)
3000–5000 yuan	172 (33.53)	84.46 ± 21.02	< 0.001	-7.40 (-11.22 ∼ -3.59)
> 5000 yuan	147 (28.65)	82.51 ± 19.93	< 0.001	-9.35 (-13.34 ∼ -5.37)

FRAS: Family Resilience Scale; SD: standard deviation; CI: Confidence Interval

Among stroke survivor characteristics, gender did not significantly affect family resilience (male: 87.62 ± 18.55; female: 85.69 ± 19.47; *p* = 0.252). There were no significant differences in family resilience across age groups (*p* > 0.05). Stroke survivors with higher education levels had significantly higher family resilience than those with junior high school education or below (93.78 ± 15.96 vs. 85.81 ± 18.35; *p* = 0.015). Stroke survivors with a shorter time since diagnosis (< 1 month) had significantly higher family resilience than those with a longer diagnosis time (89.55 ± 17.15 vs. 83.67 ± 19.87; *p* = 0.005).

Among caregiver characteristics, caregivers aged 41–60 had significantly higher family resilience than those aged 18–40 (90.46 ± 15.88 vs. 84.21 ± 19.64; *p* = 0.002). Caregivers with higher incomes (> 5,000 yuan) had significantly lower family resilience than those with lower incomes (83.33 ± 19.38 vs. 92.82 ± 12.90; *p* < 0.001) (Table 2).

**Table 2:** Demographic and clinical characteristics of caregivers with family resilience

Variable	*n* (%)	FRAS, Mean ± SD	*P*	β (95%CI)
**Gender**				
Male	221 (43.08)	85.19 ± 19.76		0.00 (Reference)
Female	292 (56.92)	87.84 ± 18.36	0.118	2.65 (-0.66 ∼ 5.97)
**Age(years)**				
18–40	212 (41.33)	84.21 ± 19.64		0.00 (Reference)
41–60	153 (29.82)	90.46 ± 15.88	0.002	6.25 (2.33 ∼ 10.17)
61–80	123 (23.98)	86.49 ± 20.87	0.287	2.28 (-1.91 ∼ 6.47)
> 80	25 (4.87)	85.88 ± 18.82	0.675	1.67 (-6.15 ∼ 9.50)
**Education**				
Elementary School	67 (13.06)	89.10 ± 19.23		0.00 (Reference)
Junior High School	139 (27.10)	85.99 ± 17.52	0.269	-3.11 (-8.62 ∼ 2.40)
High School	110 (21.44)	90.38 ± 20.12	0.663	1.28 (-4.46 ∼ 7.02)
College and Above	197 (38.40)	84.32 ± 19.02	0.074	-4.78 (-10.02 ∼ 0.46)
**Residence**				
City	335 (65.30)	86.37 ± 19.78		0.00 (Reference)
Village	178 (34.70)	87.33 ± 17.49	0.587	0.96 (-2.50 ∼ 4.42)
**Employment status**				
Employed	275 (53.61)	86.31 ± 19.15		0.00 (Reference)
Resigned	67 (13.06)	88.19 ± 17.45	0.468	1.88 (-3.20 ∼ 6.97)
Retired	171 (33.33)	86.74 ± 19.41	0.815	0.43 (-3.20 ∼ 4.07)
**Experience**				
Yes	247 (48.15)	87.86 ± 20.31		0.00 (Reference)
No	266 (51.85)	85.62 ± 17.67	0.182	-2.24 (-5.53 ∼ 1.05)
**Number of caregivers that families can share**				
Zero	114 (22.22)	87.69 ± 18.36		0.00 (Reference)
1 person	181 (35.28)	88.14 ± 19.70	0.843	0.45 (-4.00 ∼ 4.90)
2 persons	175 (34.11)	85.11 ± 18.29	0.26	-2.58 (-7.06 ∼ 1.90)
≥ 3 persons	43 (8.38)	84.44 ± 20.43	0.339	-3.25 (-9.92 ∼ 3.41)
**Monthly income (RMB)**				
< 3000 yuan	124 (24.17)	92.82 ± 12.90		0.00 (Reference)
3000–5000 yuan	181 (35.28)	86.38 ± 21.03	0.003	-6.44 (-10.71 ∼ -2.17)
> 5000 yuan	208 (40.55)	83.33 ± 19.38	< 0.001	-9.50 (-13.65 ∼ -5.34)
**Health status**				
Health	251 (48.93)	85.03 ± 19.35		0.00 (Reference)
Normal	234 (45.61)	88.12 ± 18.24	0.074	3.09 (-0.29 ∼ 6.47)
Poor	28 (5.46)	89.79 ± 21.39	0.209	4.75 (-2.65 ∼ 12.16)
**Total length of care**				
< 2 weeks	341 (66.47)	86.51 ± 17.95		0.00 (Reference)
2–3 weeks	73 (14.23)	90.38 ± 19.60	0.114	3.87 (-0.92 ∼ 8.67)
4–5 weeks	49 (9.55)	85.35 ± 20.31	0.688	-1.16 (-6.85 ∼ 4.52)
≥ 6 weeks	50 (9.75)	83.94 ± 23.23	0.372	-2.57 (-8.20 ∼ 3.06)
**Average daily care time**				
≤ 4 h	215 (41.91)	86.00 ± 20.05		0.00 (Reference)
5–8 h	187 (36.45)	85.42 ± 20.26	0.759	-0.58 (-4.30 ∼ 3.13)
> 8 h	111 (21.64)	90.20 ± 13.73	0.059	4.19 (-0.15 ∼ 8.53)
**Sleep state**				
Good	66 (12.87)	92.24 ± 19.92		0.00 (Reference)
Normal	243 (47.37)	86.47 ± 17.81	0.028	-5.78 (-10.92 ∼ -0.63)
Poor	204 (39.77)	85.19 ± 19.83	0.009	-7.06 (-12.30 ∼ -1.81)
**Chronic Disease**				
Yes	201 (39.18)	84.82 ± 20.18		0.00 (Reference)
No	312 (60.82)	87.91 ± 18.13	0.072	3.09 (-0.27 ∼ 6.45)

FRAS: Family Resilience Scale; SD: standard deviation; CI: Confidence Interval

Table 3 presents descriptive statistics for three psychological scales: FRAS-C, PSSS, and FCTI. FRAS-C averaged (86.70 ± 18.94) and average item score (2.71 ± 0.59), PSSS average item score (3.63 ± 1.09), FCTI average item score (3.57 ± 0.86), while adjusting life to caregiving needs (Subscale 5) was the lowest (3.30 ± 1.12).

**Table 3:** Descriptive statistics of FRAS-C, PSSS, and FCTI

Variable	Maximum Value	Minimum Value	Scale Score	Number of Items	Average Item Score
**FRAS-C**	128	49	86.70 ± 18.94	32	2.71 ± 0.59
USR	12	5	8.91 ± 1.99	3	2.97 ± 0.66
MPA	24	6	16.43 ± 5.49	6	2.74 ± 0.92
FCPS	92	34	61.35 ± 12.33	23	2.67 ± 0.54
**PSSS**	84	12	43.62 ± 13.14	12	3.63 ± 1.09
**FCTI**	125	54	89.26 ± 21.49	25	3.57 ± 0.86
Subscale 1	25	10	17.92 ± 4.43	5	3.58 ± 0.89
Subscale 2	25	8	18.09 ± 5.25	5	3.62 ± 1.05
Subscale 3	25	9	18.51 ± 4.74	5	3.70 ± 0.95
Subscale 4	25	5	18.23 ± 4.82	5	3.65 ± 0.96
Subscale 5	25	5	16.51 ± 5.62	5	3.30 ± 1.12

FRAS-C: Family Resilience Assessment Scale, USR: Utilization of Social Resources

MPA: aintaining a Positive Attitude, FCPS: Family Communication and Problem-Solving

PSSS: The Perceived Social Support Scale; FCTI: Family Caregiver Training Inventory

Subscale 1: Adapting to the caregiver role

Subscale 2: Responding flexibly to provide care

Subscale 3: Managing personal emotions

Subscale 4: Assessing family and community resources

Subscale 5: Adjusting life to meet caregiving needs

Table 4 presents the key factors that influence the resilience of stroke survivors’ families. Higher education levels (college and above: β = 8.50, 95% CI = 2.27–14.73, *p* = 0.008) and mild dependence in ADL: β = 9.40, 95% CI = 4.57–14.23, *p* < 0.001) were positively associated with family resilience. Conversely, Self-financing coverage (β = -6.47, 95% CI = -11.30–1.65, *p* = 0.009), higher pre-illness income of stroke survivors (> 5000 yuan: β = -8.31, 95% CI = -13.43–3.20, *p* = 0.002), and suboptimal caregiver sleep quality (normal/poor vs. good: both β = -6.20, *p* < 0.05) demonstrated negative associations.

**Table 4:** Multivariate linear regression analysis the factors influencing family resilience

Variables	β	S.E	t	*P*	β (95%CI)
**Intercept**	97.73	3.35	29.21	< 0.001	97.73 (91.17 ∼ 104.29)
**Education of stroke survivors**					
Junior High Schoo					0.00 (Reference)
High School	2.29	1.93	1.19	0.236	2.29 (-1.50 ∼ 6.07)
College and Above	8.5	3.18	2.68	0.008	8.50 (2.27 ∼ 14.73)
**Medical of stroke survivors**					
Rural medical insurance					0.00 (Reference)
Urban medical insurance	0.28	1.93	0.15	0.883	0.28 (-3.51 ∼ 4.08)
Commercial insurance	-1.27	3.04	-0.42	0.676	-1.27 (-7.22 ∼ 4.68)
Self-financing	-6.47	2.46	-2.63	0.009	-6.47 (-11.30 ∼ -1.65)
**ADL of stroke survivors**					
Moderate dependence					0.00 (Reference)
Severe dependence	-4.25	1.94	-2.19	0.029	-4.25 (-8.04 ∼ -0.45)
Mild dependence	9.4	2.47	3.81	< 0.001	9.40 (4.57 ∼ 14.23)
**The pre-illness monthly income of stroke survivors (RMB)**					
< 3000 yuan					0.00 (Reference)
3000–5000 yuan	-5.34	2.49	-2.14	0.033	-5.34 (-10.22 ∼ -0.46)
> 5000 yuan	-8.31	2.61	-3.19	0.002	-8.31 (-13.43 ∼ -3.20)
**Sleep state state of caregiver**					
Good					0.00 (Reference)
Normal	-6.2	2.61	-2.37	0.018	-6.20 (-11.32 ∼ -1.07)
Poor	-6.2	2.67	-2.32	0.021	-6.20 (-11.43 ∼ -0.97)

CI: Confidence Interval

### Correlation analysis

The Pearman correlation analysis results showed that FRAS-C was significantly positively correlated with PSSS (*r* = 0.523, *p* < 0.01) and FRAS-C was significantly positively correlated with FCTI (*r* = 0.522, *p* < 0.01). PSSS was also positively correlated with FCTI (*r* = 0.377, *p* < 0.01) (Table 5).

**Table 5:** Correlations (r) between FRAS-C, PSSS, and FCTI

	PSSS	FRAS-C	FCTI
PSSS	1		
FRAS-C	0.523**	1	
FCTI	0.377**	0.522**	1

The Family Resilience Scale (FRAS.C) assesses family resilience

The Family Caregiver Task lnventory (FCTl) assesses caregiver capacity

The Perceived Social Support Scale (PSSS) assess an individuals perceivedlevel of social support. **p-value < 0.01

### Mediation analysis

**Fig. 1:**
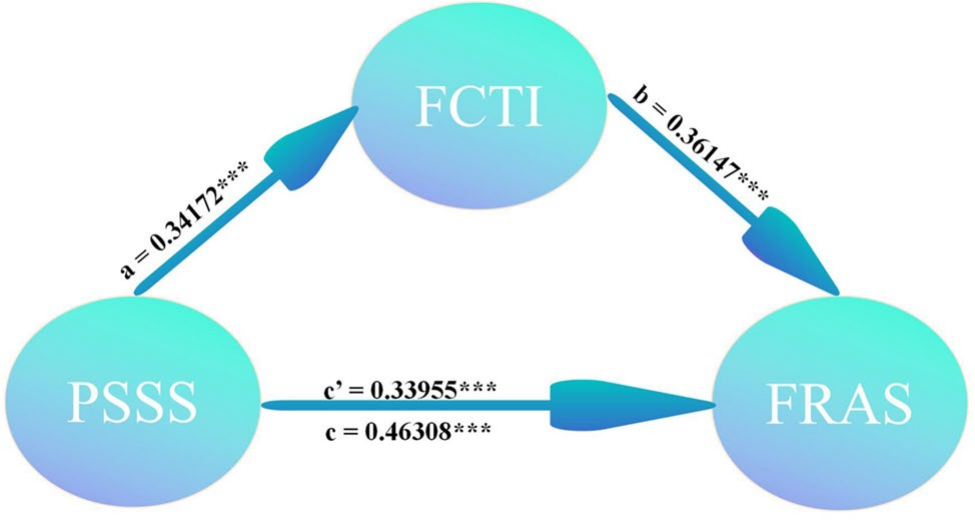
Model of the mediating effects of caregiver competence on social support and family resilience

Figure 1 illustrates the mediating effects of FCTI on the relationship between PSSS and FRAS-C. The percentile bootstrap method for bias correction was adopted to test the mediating effect at a 95% confidence interval. Five thousand samples were repeatedly and randomly selected from the original samples for model fitting.

Table 6 indicates that FCTI significantly mediates the relationship between PSSS and FRAS-C. The indirect effect of PSSS on FRAS-C through FCTI was 0.1784 (95% CI: 0.130 to 0.2293, *p* < 0.001), accounting for approximately 26.68% of the total effect. The direct effect of PSSS on FRAS was 0.4904 (95% CI: 0.374 to 0.597, *p* < 0.001), representing 73.32% of the total effect. The total effect of PSSS on FRAS was 0.6688 (95% CI: 0.56 to 0.7716, *p* < 0.001).

**Table 6:** The mediating effect of FCTI on the association between PSSS and FRAS-C

Type	Effect	Estimate	SE	95% C.I. (a)		β	z	*p*
				Lower	Upper			
Indirect	PSSS ⇒ FCTI ⇒ FRAS	0.1784	0.0246	0.1310	0.2307	0.12353	7.2486	< 0.001
Component	PSSS ⇒ FCTI	0.5582	0.0586	0.4450	0.6792	0.34172	9.5280	< 0.001
	FCTI ⇒ FRAS	0.3196	0.0332	0.2530	0.3838	0.36147	9.6205	< 0.001
Direct	PSSS ⇒ FRAS	0.4904	0.0550	0.3820	0.5970	0.33955	8.9242	< 0.001
Total	PSSS ⇒ FRAS	0.6688	0.0532	0.5670	0.7746	0.46308	12.5810	< 0.001

The path analysis revealed that PSSS had a significant positive effect on FCTI (β = 0.34172, *p* < 0.001), indicating that higher perceived social support is associated with greater caregiver capacity. Additionally, FCTI had a significant positive effect on FRAS-C (β = 0.36147, *p* < 0.001), suggesting that lower caregiver capacity is associated with reduced family resilience.

## Discussion

An integrated analysis of this study identified significant factors affecting family resilience in stroke survivors, with a particular emphasis on mediation effects. The findings indicated that caregiver capacity mediates the relationship between PSS and family resilience. This underscores the intricate interactions within family systems and is consistent with global research highlighting the psychosocial influences on family resilience [38, 39].

### Sociodemographic, socioeconomic, and clinical factors associated with family resilience in stroke survivors

The demographic and clinical characteristics of stroke survivors and their caregivers. It reveals the relationship between these characteristics and family resilience.

Among stroke survivors, several characteristics are significantly associated with family resilience. Stroke survivors with shorter time since diagnosis (< 1 month) had higher family resilience, suggesting that early diagnosis and intervention may enhance a family’s ability to cope with illness [40, 41]. Higher education levels were associated with greater family resilience, likely due to better access to resources, problem-solving skills, and adaptive coping mechanisms [42]. The positive association between advanced education and resilience aligns with cognitive reserve theories, which suggest that higher educational attainment may enhance problem-solving capacities in caregiving scenarios [43]. Additionally, ADL is significantly associated with family resilience. Mild dependence usually requires less care and support. This dependence may be related to a reduction in the burden on caregivers and a higher level of family resilience. Severe dependence is associated with an increase in the burden on caregivers and a lower level of family resilience. The importance of functional independence of stroke survivors in maintaining family stability was emphasized [44]. In terms of medical payment methods, the type of medical insurance is an important related factor for family resilience. Given that stroke rehabilitation can impose a significant economic burden on families, medical insurance is believed to alleviate this pressure by providing both economic and social support [24], and its role is crucial for family resilience. Therefore, the government needs to formulate relevant strategies to reduce the economic burden.

An interesting finding is that having a relatively high income level (> 5,000 yuan) before the onset of stroke may have adverse effects, which contradicts the traditional theory of resource accumulation [45]. This finding might stem from the loss of status, career interruption, and role upheaval experienced by the survivors [46], especially when they suddenly shift from being the primary breadwinner of their families to relying on others for care. This drastic psychological shift significantly increases the difficulty of rebuilding family resilience. At the same time, when survivors care, the primary caregivers, who are usually family members, are often forced to suspend their work, such as reducing working hours, taking leave, or quitting, further intensifying the economic pressure on the families. The heavy burden of care also makes it difficult for caregivers to maintain social relationships, leaving them in a state of isolation [47]. The experience of family role transformation and the excessive burden on caregivers can undermine effective communication within the family, thereby weakening the family’s overall resilience.

Among caregivers, age, income, and sleep quality are associated with family resilience. Middle-aged caregivers (41–60 years) may exhibit a higher level of family resilience, which may be related to their more mature life experiences and stronger coping abilities. Meanwhile, caregivers with lower incomes had higher family resilience, possibly due to their greater reliance on family support. Furthermore, the sleep state is a key factor related to this. Poorer sleep quality is significantly associated with a lower level of family resilience. This aligns with previous research indicating that caregiver well-being is closely tied to family functioning [48]. Interventions to improve caregiver sleep quality, such as respite care or stress management programs, could enhance family resilience.

### The mediating role of FCTI

This study explores the interplay between PSSS, family resilience (FRAS-C), and caregiver capacity. It underscores the importance of PSSS in enhancing family resilience, both directly (β = 0.33955, *p* < 0.001) and indirectly (β = 0.12353, 95% CI: 0.13–0.2293). This finding suggests that families with stronger social support networks are better equipped to cope with challenges, improving their resilience. This aligns with previous research indicating that social support is a buffer against stress and enhances adaptive coping mechanisms [49–51]. Conceptually, social support can be viewed as a critical resource that empowers families to mobilize internal and external resources to address challenges effectively. This perspective is consistent with the Family Resilience Framework [52, 53], which posits that resilience is a dynamic process shaped by the interplay of risk and protective factors. Within this framework, social support is a protective factor that buffers the adverse effects of caregiving stress and enhances the family’s adaptive capacity. Furthermore, our findings support the Social-Ecological Model [27], which emphasizes the pivotal role of social networks in shaping health outcomes. By integrating these theoretical perspectives, our study provides a comprehensive framework for understanding how social support contributes to family resilience. In summary, the study highlights the critical role of PSSS as a fundamental pillar of family resilience in stroke care, underscoring the need to establish support networks for families facing challenges. These findings offer a strong foundation for developing targeted intervention strategies and policy-making.

Investigations indicate that FCTI is mediating between PSSS and FRAS-C. Lower caregiver capacity is associated with reduced family resilience, consistent with prior work by Xu et al. [22]. Our findings suggest that lower caregiving ability may indicate that family members require more support and resources, which in turn impairs their ability to cope with stress, thereby affecting family resilience. This finding supports the Social Support Theory, which posits that the social support system influences an individual’s ability to cope in stressful situations [54, 55]. This theoretical perspective offers new insights into understanding family resilience, particularly in the caregiving role, where family resilience is contingent upon individual coping mechanisms and influenced by caregiving ability. In line with the Family Resilience Framework [23]. This study found that caregivers demonstrated low capabilities in role adaptation and life-caregiving balance. We recommend implementing structured role adaptation programs to help caregivers establish realistic expectations, set boundaries, and develop healthy coping strategies. Additionally, personalized life integration support, including respite care and time management training, can improve their ability to balance personal and caregiving responsibilities. The role of social support in enhancing caregiver capacity suggests that it can provide additional resources. For instance, policymakers can enhance support for resources and mental health interventions for families with lower caregiving abilities. These interventions will enhance both individual well-being and overall family resilience.

### Limitations

This study has several limitations. First, the cross-sectional design precludes causal inferences regarding temporal relationships among PSS, caregiver capacity, and family resilience. Directionality remains ambiguous, whether PSS enhances caregiver capacity or resilient families attract more support. Second, convenience sampling from two urban hospitals in Ningbo constrains generalizability beyond inpatient settings and risks selection bias, particularly as our hospital-based recruitment excluded severe cases requiring ICU/rehabilitation care, potentially limiting generalizability to families of critically ill patients in the acute phase. Crucially, this hospital-centric approach overlooks post-discharge challenges. Findings may not reflect resilience dynamics during home-based care transitions, particularly in community contexts. Third, exclusive caregiver self-reports for all constructs (PSSS, FCTI, FRAS-C) introduce mono-informant bias and potential shared method variance; objective assessments were absent. Correlations may be artificially inflated, compromising the validity of mediation pathways. Finally, the absence of stroke survivor perspectives restricts holistic family-system understanding. Critical dyadic interactions within resilience processes remain unexamined.

## Conclusion

This study advances prior research by systematically examining the mediating role of caregiver capacity in linking PSS to family resilience among stroke survivors, providing new insights into family resilience among this population. First, we identified that higher pre-stroke income may paradoxically be associated with lower family resilience, challenging conventional resource accumulation theories and highlighting the understudied psychological impact of sudden role reversal in higher-income households. Second, our mediation analysis provides empirical evidence that caregiver capacity serves as a crucial pathway through which social support benefits family resilience, offering a more nuanced understanding than previous direct-effect models. Third, we identified sleep quality as a modifiable caregiver factor associated with resilience, suggesting new intervention targets beyond the more commonly studied socioeconomic factors. Although constrained by the cross-sectional design, these findings enhance our understanding of family adaptation following stroke. They can guide better support programs that combine social networks with caregiver training. Future research should track families over time and include home care settings to understand the impact of these interventions.

## Supplementary Information

Below is the link to the electronic supplementary material.

Supplementary Material 1

## Data Availability

The datasets generated and analysed during the current study are not publicly available because they contain private information about hospitals and patients, but they are available from the corresponding author on reasonable request.

## Declarations

### Patient and public involvement

Patients and/or the public were not involved in the design, or conduct, or reporting, or dissemination plans of this research.

## Abbreviations

PSSS: Perceived Social Support Scale
FCTI: Family Caregiver Task Inventory
FRAS-C: Family Resilience Assessment Scale
ADL: Activities of Daily Living

## References

[1] GBD 2019 Stroke Collaborators. Global, regional, and National burden of stroke and its risk factors, 1990–2019: a systematic analysis for the global burden of disease study 2019. Lancet Neurol. 2021;20(10):795–820. 10.1016/S1474-4422(21)00252-0. 34487721 10.1016/S1474-4422(21)00252-0 PMC8443449

[2] Wang YJ, Li ZX, Gu HQ, Zhai Y, Jiang Y, Zhou Q, et al. China stroke statistics 2020 (Chinese version)(1). Zhongguo Zu Zhong Za Zhi [Chinese J Stroke]. 2022;17(5):433–47.

[3] Potter TBH, Tannous J, Vahidy FS. A contemporary review of epidemiology, risk factors, etiology, and outcomes of premature stroke. Curr Atheroscler Rep. 2022;24(12):939–48. 10.1007/s11883-022-01067-x. 36374365 10.1007/s11883-022-01067-x PMC9660017

[4] Lu Q, Mårtensson J, Zhao Y, Johansson L. Living on the edge: family caregivers’ experiences of caring for post-stroke family members in china: A qualitative study. Int J Nurs Stud. 2019;94:1–8. 10.1016/j.ijnurstu.2019.02.016. 30928717 10.1016/j.ijnurstu.2019.02.016

[5] Mooney-Doyle K, Dos Santos MR, Szylit R, Deatrick JA. Parental expectations of support from healthcare providers during pediatric life-threatening illness: a secondary, qualitative analysis. J Pediatr Nurs. 2017;36:163–72. 10.1016/j.pedn.2017.05.008 10.1016/j.pedn.2017.05.008 PMC5659330 28888498

[6] Walsh F. Applying a family resilience framework in training, practice, and research: mastering the Art of the possible. Fam Process. 2016;55(4):616–32. 10.1111/famp.12260. 27921306 10.1111/famp.12260

[7] Wang Y, Xie H, Sun H, Ren L, Jiang H, Chen M, Dong C. Influencing factors of psychological resilience in stroke patients: A systematic review and Meta-Analysis. Arch Clin Neuropsychol. 2024;39(5):644–54. 10.1093/arclin/acad107. 38324660 10.1093/arclin/acad107

[8] Zhang W, Ye MM, Gao YJ, Zhou LS. Dyadic profiles of family resilience among patients with first-episode stroke: A longitudinal study of the first 6 months after stroke. J Clin Nurs. 2023;32(13–14):3672–81. 10.1111/jocn.16458. 35864722 10.1111/jocn.16458

[9] Han K, Chen Y, Li M, Cui L. Using a Mixed-Method approach to explore the factors influencing the family resilience of stroke survivors in China. J Multidiscip Healthc. 2024;17:275–87. 10.2147/JMDH.S439737. 38264410 10.2147/JMDH.S439737 PMC10804964

[10] Sarason BR, Sarason IG, Pierce GR. Traditional views of social support and their impact on assessment. In: Sarason BR, Sarason IG, Pierce GR, editors. Social support: an interactional view. New York: John Wiley & Sons; 1990. p. 9–25.

[11] Wang H, Zhu L, Cao W, Yang W, Gao Y, Yao G, Zhang H, Li G. Family resilience, patient-reported symptoms in young stroke dyads: the effect of caregiver readiness and social support. J Clin Nurs. 2024;33(10):3954–68. 10.1111/jocn.17046. 38348545 10.1111/jocn.17046

[12] Wang J, Mann F, Lloyd-Evans B, Ma R, Johnson S. Associations between loneliness and perceived social support and outcomes of mental health problems: a systematic review. BMC Psychiatry. 2018;18(1):156. 10.1186/s12888-018-1736-5. 29843662 10.1186/s12888-018-1736-5 PMC5975705

[13] Farran CJ, McCann JJ, Fogg LG, Etkin CD. Developing a measurement strategy for assessing family caregiver skills: conceptual issues. Alzheimers Care Today. 2009;10(3):129–39. 10.1097/ACQ.0b013e3181b15d82. 20179779 10.1097/ACQ.0b013e3181b15d82 PMC2825897

[14] LeLaurin JH, Lamba AH, Eliazar-Macke ND, Schmitzberger MK, Freytes IM, Dang S, et al. Postdischarge intervention for stroke caregivers: protocol for a randomized controlled trial. JMIR Res Protoc. 2020;9(11):e21799. 10.2196/21799. 33174856 10.2196/21799 PMC7688383

[15] Cheng HY, Chair SY, Chau JPC. Effectiveness of a strength-oriented psychoeducation on caregiving competence, problem-solving abilities, psychosocial outcomes and physical health among family caregiver of stroke survivors: A randomised controlled trial. Int J Nurs Stud. 2018;87:84–93. 10.1016/j.ijnurstu.2018.07.005. 30059815 10.1016/j.ijnurstu.2018.07.005

[16] Braveman P, Gottlieb L. The social determinants of health: it’s time to consider the causes of the causes. Public Health Rep. 2014;129(2):19–31. 10.1177/00333549141291S206. 10.1177/00333549141291S206 PMC3863696 24385661

[17] De Vogli R, Gimeno D, Kivimaki M. Socioeconomic inequalities in health in 22 European countries. N Engl J Med. 2008;359(12):1290. 10.1056/NEJMc081414. author reply 1290-1. 18799564 10.1056/NEJMc081414

[18] Sposato LA, Saposnik G. Gross domestic product and health expenditure associated with incidence, 30-day fatality, and age at stroke onset: a systematic review. Stroke. 2012;43(1):170–7. 10.1161/STROKEAHA.111.632158. 22033985 10.1161/STROKEAHA.111.632158

[19] Chen DD, Zha Y, Wang Q, Sha S, Luo YR, Zhang Y, et al. Relationship between social support and family resilience in primary caregivers of first-episode stroke patients: a chain mediation model. J Nav Med Univ. 2025;46(4):451–7. 10.16781/j.CN31-2187/R.20250023.

[20] Wang S, Lu Q, Zhang D, Wang L, Jin H, Zhou Y, Ma R. Mediation effect of self-efficacy on the relationship between perceived social support and resilience in caregivers of patients with first-stroke in china: a cross-sectional survey. Top Stroke Rehabil. 2024;31(6):595–603. 10.1080/10749357.2024.2318087. 38375811 10.1080/10749357.2024.2318087

[21] Lu Y, Yan L. Impact of perceived social support on family resilience in patients with ischemic stroke: a mediation model analysis. Geriatr Nurs. 2024;60:456–61. 10.1016/j.gerinurse.2024.10.004 10.1016/j.gerinurse.2024.10.004 39423577

[22] Xu Q, Ge Q, Shi L, Zhang Y, Ma J. Assessing the mediating role of family resilience between caregiver burden and caregiver capacity: a cross-sectional study among Chinese stroke survivors and family caregivers in a real-world setting. BMJ Open. 2024;14(5):e083106. 10.1136/bmjopen-2023-083106. 38724057 10.1136/bmjopen-2023-083106 PMC11086420

[23] Walsh F. Family resilience: a framework for clinical practice. Fam Process. 2003;42(1):1–18. 10.1111/j.1545-5300.2003.00001.x. 10.1111/j.1545-5300.2003.00001.x 12698595

[24] Ye Q, Yang Y, Li J, Wang T, Liu N. How does family resilience develop among stroke survivors and their caregivers? A mixed-method study using a chain mediating model. Int J Nurs Stud Adv. 2024;7:100246. 10.1016/j.ijnsa.2024.100246. 39391565 10.1016/j.ijnsa.2024.100246 PMC11465215

[25] Blok AC, Gauntlett L, Jayaram M, Krein SL. Caregiver and care team perspectives of caregiver psychological distress and well-being during critical care hospitalization: a qualitative study. BMC Geriatr. 2025;25(1):167. 10.1186/s12877-025-05769-0. 40082775 10.1186/s12877-025-05769-0 PMC11905728

[26] Zhang W, Gao YJ, Ye MM, Zhou LS. Post-stroke family resilience is correlated with family functioning among stroke survivors: the mediating role of patient’s coping and self-efficacy. Nurs Open. 2024;11(7):e2230. 10.1002/nop2.2230. 38940513 10.1002/nop2.2230 PMC11212063

[27] Holt-Lunstad J. Why social relationships are important for physical health: A systems approach to Understanding and modifying risk and protection. Annu Rev Psychol. 2018;69:437–58. 10.1146/annurev-psych-122216-011902. 29035688 10.1146/annurev-psych-122216-011902

[28] Chandran D, Corbin JH, Shillam C. An ecological Understanding of caregiver experiences in palliative care. J Soc Work End Life Palliat Care. 2016;12(1–2):162–82. 10.1080/15524256.2016.1156602 10.1080/15524256.2016.1156602 27143579

[29] Cui P, Yang M, Hu H, Cheng C, Chen X, Shi J, Li S, Chen C, Zhang H. The impact of caregiver burden on quality of life in family caregivers of patients with advanced cancer: a moderated mediation analysis of the role of psychological distress and family resilience. BMC Public Health. 2024;24(1):817. 10.1186/s12889-024-18321-3. 38491454 10.1186/s12889-024-18321-3 PMC10941369

[30] Xiao C, Patrician PA, Montgomery AP, Wang Y, Jablonski R, Markaki A. Filial piety and older adult caregiving among Chinese and Chinese-American families in the united states: a concept analysis. BMC Nurs. 2024;23(1):115. 10.1186/s12912-024-01789-0. 38347512 10.1186/s12912-024-01789-0 PMC10863110

[31] Denham AMJ, Baker AL, Spratt N, Guillaumier A, Wynne O, Turner A, Magin P, Bonevski B. The unmet needs of informal carers of stroke survivors: a protocol for a systematic review of quantitative and qualitative studies. BMJ Open. 2018;8(1):e019571. 10.1136/bmjopen-2017-019571. 29391371 10.1136/bmjopen-2017-019571 PMC5878248

[32] Preacher KJ, Kelley K. Effect size measures for mediation models: quantitative strategies for communicating indirect effects. Psychol Methods. 2011;16(2):93–115. 10.1037/a0022658. 21500915 10.1037/a0022658

[33] Mlinac ME, Feng MC. Assessment of activities of daily living, Self-Care, and independence. Arch Clin Neuropsychol. 2016;31(6):506–16. 10.1093/arclin/acw049. 27475282 10.1093/arclin/acw049

[34] Chow TS, Tang CSK, Siu TSU, Kwok HSH. Family resilience scale short form (FRS16): validation in the US and Chinese samples. Front Psychiatry. 2022;13:845803. 10.3389/fpsyt.2022.845803. 35633805 10.3389/fpsyt.2022.845803 PMC9136042

[35] Zimet GD, Powell SS, Farley GK, Werkman S, Berkoff KA. Psychometric characteristics of the multidimensional scale of perceived social support. J Pers Assess 1990;55(3–4):610–7. 10.1080/00223891 10.1080/00223891.1990.9674095 2280326

[36] Liu L, Gou Z, Zuo J. Social support mediates loneliness and depression in elderly people. J Health Psychol. 2016;21(5):750–8. 10.1177/1359105314536941. 24925547 10.1177/1359105314536941

[37] Lee RL, Mok ES. Evaluation of the psychometric properties of a modified Chinese version of the caregiver task Inventory–refinement and psychometric testing of the Chinese caregiver task inventory: a confirmatory factor analysis. J Clin Nurs. 2011;20(23–24):3452–62. 10.1111/j.1365-2702. 21707805 10.1111/j.1365-2702.2011.03729.x

[38] Kim GM, Lim JY, Kim EJ, Park SM. Resilience of patients with chronic diseases: A systematic review. Health Soc Care Community. 2019;27(4):797–807. 10.1111/hsc.12620. 30027595 10.1111/hsc.12620

[39] Qureshi A, Hargest C, Swain N, Aldabe D, Hale L. Psychosocial interventions for Building resilience of informal carers of people living with stroke: a systematic review. Disabil Rehabil. 2023;45(9):1419–32. 10.1080/09638288.2022.2063419. 35468030 10.1080/09638288.2022.2063419

[40] Mou H, Lam SKK, Chien WT. The effects of a family-focused dyadic psychoeducational intervention for stroke survivors and their family caregivers: A randomised controlled trial. Int J Nurs Stud. 2023;143:104504. 10.1016/j.ijnurstu.2023.104504. 37149953 10.1016/j.ijnurstu.2023.104504

[41] Mou H, Wong MS, Chien WT, Prof. Effectiveness of dyadic psychoeducational intervention for stroke survivors and family caregivers on functional and psychosocial health: A systematic review and meta-analysis. Int J Nurs Stud. 2021;120:103969. 10.1016/j.ijnurstu.2021.103969. 34052538 10.1016/j.ijnurstu.2021.103969

[42] Lu Y. Independent predictors of family resilience in patients with ischemic stroke: A cross-sectional survey. Heliyon. 2024;10(3):e25062. 10.1016/j.heliyon.2024.e25062. 38317932 10.1016/j.heliyon.2024.e25062 PMC10839963

[43] Stern Y. What is cognitive reserve? Theory and research application of the reserve concept. J Int Neuropsychol Soc. 2002;8(3):448–60. 10.1017/S1355617702813248. 11939702

[44] Xu Q, Ma J, Zhang Y, Gan J. Family resilience and social support as mediators of caregiver burden and capacity in stroke caregivers: a cross-sectional study. Front Psychol. 2024;15:1435867. 10.3389/fpsyg.2024.1435867. 39698379 10.3389/fpsyg.2024.1435867 PMC11653184

[45] Iskandar M, Martindale J, Bynum JPW, Davis MA. Association between family household income and cognitive resilience among older US adults: A Cross-Sectional study. J Prev Alzheimers Dis. 2024;11(5):1406–9. 10.14283/jpad.2024.97. 39350387 10.14283/jpad.2024.97 PMC12598677

[46] Orange C, Lanhers C, Coll G, Coste N, Dutheil F, Hauret I, Pereira B, Coudeyre E. Determinants of return to work after a stroke: A systematic review and Meta-analysis. Arch Phys Med Rehabil. 2024;105(2):359–68. 10.1016/j.apmr.2023.08.027. 37797913 10.1016/j.apmr.2023.08.027

[47] Adelman RD, Tmanova LL, Delgado D, Dion S, Lachs MS. Caregiver burden: a clinical review. JAMA. 2014;311(10):1052–60. 10.1001/jama.2014.304. 24618967 10.1001/jama.2014.304

[48] Wang X, Wang RX, Bian C, Liu FY, Tang MW, Zhang YH. Sleep quality, psychological resilience, family resilience, social support, and mental disability in patients with chronic schizophrenia: A cross-sectional study. Schizophr Res. 2024;274:199–205. 10.1016/j.schres.2024.09.020. 39341099 10.1016/j.schres.2024.09.020

[49] Last BS, Triplett NS, McGinty EE, Waller CR, Khazanov GK, Beidas RS. The social determinants of resilience: A conceptual framework to integrate psychological and policy research. Am Psychol. 2024;79(8):1049–62. 10.1037/amp0001308. 39531706 10.1037/amp0001308 PMC11889538

[50] Thoits PA. Mechanisms linking social ties and support to physical and mental health. J Health Soc Behav. 2011;52(2):145–61. 10.1177/0022146510395592. 21673143 10.1177/0022146510395592

[51] Tindle R, Hemi A, Moustafa AA. Social support, psychological flexibility and coping mediate the association between COVID-19 related stress exposure and psychological distress. Sci Rep. 2022;12(1):8688. 10.1038/s41598-022-12262-w. 35606392 10.1038/s41598-022-12262-w PMC9126245

[52] Yu Y, Li D, Xia Y. Applying kumpfer’s resilience framework to understand the social adaptation process of the trailing parents in China. BMC Geriatr. 2024;24(1):587. 10.1186/s12877-024-05170-3. 38982345 10.1186/s12877-024-05170-3 PMC11232334

[53] Hynes L, Saetes S, McGuire B, Caes L. Child and family adaptation to juvenile idiopathic Arthritis-A systematic review of the role of resilience resources and mechanisms. Front Psychol. 2019;10:2445. 10.3389/fpsyg.2019.02445. 31749744 10.3389/fpsyg.2019.02445 PMC6848885

[54] Zhao YC, Zhao M, Song S. Online health information seeking among patients with chronic conditions: integrating the health belief model and social support theory. J Med Internet Res. 2022;24(11):e42447. 10.2196/42447. 36322124 10.2196/42447 PMC9669891

[55] Rook KS. Social support versus companionship: effects on life stress, loneliness, and evaluations by others. J Pers Soc Psychol. 1987;52(6):1132–47. 10.1037//0022-3514.52.6.1132. 10.1037//0022-3514.52.6.1132 3598859

