# Supplementary material for: Perceived social support, caregiver capacity, and socioeconomic determinants mediating pathways to family resilience in Chinese stroke survivors: a cross-sectional study
Source: BMC Nurs. 2025 Sep 26;24:1180. doi: 10.1186/s12912-025-03826-y (PMC12465349; doi:10.1186/s12912-025-03826-y)
Supplement: Supplementary file 1 — Supplementary Material 1 [file 12912_2025_3826_MOESM1_ESM.docx]

**Family Resilience Questionnaire for Stroke Caregivers**

Dear caregiver,

This questionnaire aims to understand family resilience and related characteristics during your caregiving journey for stroke survivors. All information will be used solely for research purposes and kept strictly confidential. Please answer according to your actual situation. Thank you for your support!

**Demographic Information**

Gender

☐ Male

☐ Female

Age

☐ 18–40 years

☐ 41–60 years

☐ 61–80 years

☐ >80 years

Education Level

☐ Primary School

☐ Junior High School

☐ High School/Vocational School

☐ College or Higher

Residence

☐ Urban

☐ Rural

Employment Status

☐ Currently Employed

☐ Unemployed (Voluntarily Resigned)

☐ Retired

Monthly Income (RMB)

☐ <3,000 yuan

☐ 3,000–5,000 yuan

☐ >5,000 yuan

**Caregiving Experience & Health Status**

Prior Caregiving Experience

☐ Yes

☐ No

Number of Additional Family Caregivers Available

☐ None

☐ 1 person

☐ 2 people

☐ ≥3 people

Self-Rated Health Status

☐ Excellent

☐ Normal

☐ Poor

Chronic Disease Diagnosis

☐ Yes

☐ No

Sleep Quality

☐ Good

☐ Normal

☐ Poor

**Caregiving Workload**

Total Duration of Caregiving

☐ <2 weeks

☐ 2–3 weeks

☐ 4–5 weeks

☐ ≥6 weeks

Average Daily Caregiving Hours

☐ ≤4 hours

☐ 5–8 hours

☐ >8 hours

**Instructions for Completing the Family Caregiver Task Inventory (FCTI):**

The following questions are designed to understand the level of difficulty you experience while caring for the patient. There are no right or wrong answers—please respond based on your actual situation. The scoring options are: 1 = Not difficult at all，2 = Slightly difficult，3 = Neutral/Uncertain，4 = Difficult，5 = Extremely difficult .Please check (✓) the appropriate box for each item.

| **Family Caregiver Task Inventory (FCTI)** | | | | | | |
| --- | --- | --- | --- | --- | --- | --- |
| Sub-scale | May I ask how difficult it is for you to achieve the following things? | Not difficult at all | Slightly difficult | Neutral/ Uncertain | Difficult | Extremely difficult |
| 1: Adapting to the caregiver role | Monitor course of condition and evaluate significance of changes |  |  |  |  |  |
|  | Normalise care-receiver routine, within bounds of the impairment |  |  |  |  |  |
|  | Perform basic ADL for the care-receiver |  |  |  |  |  |
|  | Gain knowledge about the disease |  |  |  |  |  |
|  | Cope with the loss/restriction of future family plans |  |  |  |  |  |
| 2: Responding flexibly to provide care | Be available when needed |  |  |  |  |  |
|  | Supervise prescribed treatments and general recommendations |  |  |  |  |  |
|  | Evaluate strength/resources of the care-receiver |  |  |  |  |  |
|  | Cope with upsetting behaviour of the care-receiver |  |  |  |  |  |
|  | Give appropriate consideration to care-receiver’s options and preferences |  |  |  |  |  |
| 3: Managing personal emotions | Resolve guilt over ‘negative feelings’ towards care-receiver |  |  |  |  |  |
|  | Find a locus of blame for the condition/disease |  |  |  |  |  |
|  | Separate feelings regarding condition from feelings toward the care-receiver |  |  |  |  |  |
|  | Resolve uncertainty about one’s skills as a caregiver |  |  |  |  |  |
|  | Release tensions/feelings toward the care-receiver |  |  |  |  |  |
| 4: Assessing family and community resources | Anticipate needs for future assistance |  |  |  |  |  |
|  | Designate other responsible caregiver(s) |  |  |  |  |  |
|  | Manage feelings toward other family members who do not regularly help |  |  |  |  |  |
|  | Maintain the family as effective decision-making group over a long period of time |  |  |  |  |  |
|  | Interact with medical, health and social service professionals |  |  |  |  |  |
| 5: Adjusting life to meet caregiving needs | Satisfy needs for creativity/originality to offset tedious routine |  |  |  |  |  |
|  | Avoid severe drain on physical strength/health |  |  |  |  |  |
|  | Make up for or avoid loss/restrictions on future plans and perspectives |  |  |  |  |  |
|  | Readjust personal routine |  |  |  |  |  |
|  | Compensate for disruption of sleep |  |  |  |  |  |

**The Family Resilience Assessment Scale (FRAS)**

Evaluates resilience across three dimensions: family communication/problem-solving, social resource utilization, and positive outlook, through 32 items rated on a 4-point Likert scale (1=Strongly Disagree to 4=Strongly Agree). Total scores range from 32 to 128, with higher scores indicating greater family resilience. Please indicate how well each statement reflects your family's actual situation by checking the appropriate response. There are no correct answers - your honest assessment will help us understand your family's resilience characteristics.

| **Family Resilience Assessment Scale, FRAS-C** | | | | | |
| --- | --- | --- | --- | --- | --- |
| N0 | Item | Strongly Disagree | Disagree | Agree | Strongly Agree |
| 1 | Our family can deal with emergencies flexibly. |  |  |  |  |
| 2 | Hurt caused by improper words and deeds between family members can be quickly forgiven |  |  |  |  |
| 3 | Our family ADAPTS to the challenges that the outside world throws at us. |  |  |  |  |
| 4 | Our family is open to new ways of doing things. |  |  |  |  |
| 5 | Family members understand each other. |  |  |  |  |
| 6 | In times of trouble, we turn to our neighbors for help and support. |  |  |  |  |
| 7 | We believe we can deal with the problems we encounter. |  |  |  |  |
| 8 | We can ask each other for explanations when we don't understand each other. |  |  |  |  |
| 9 | Our family can be honest and open with each other. |  |  |  |  |
| 10 | We can lose our temper at home and get understanding from our family. |  |  |  |  |
| 11 | When family conflicts occur, family members can give in to each other to achieve compromise. |  |  |  |  |
| 12 | When it comes to bearing losses, we are able to deal with disagreements among family members. |  |  |  |  |
| 13 | We can solve big problems together. |  |  |  |  |
| 14 | Even when it rains, we can still get through it. |  |  |  |  |
| 15 | We can discuss how family members should communicate with each other. |  |  |  |  |
| 16 | As a family, we can get through this. |  |  |  |  |
| 17 | We are able to make family decisions through mutual consultation. |  |  |  |  |
| 18 | We can identify problems and solve them. |  |  |  |  |
| 19 | We can agree on family issues. |  |  |  |  |
| 20 | We were able to discuss family problems together until we came to a new solution. |  |  |  |  |
| 21 | In my family, everyone is free to express their opinions when confronted with problems. |  |  |  |  |
| 22 | We are willing to devote time and energy to solving family problems. |  |  |  |  |
| 23 | We believe that people in the community or village are willing to help in an emergency. |  |  |  |  |
| 24 | We feel that our family is strong in the face of big problems. |  |  |  |  |
| 25 | We have the ability to solve our family's problems. |  |  |  |  |
| 26 | We know that we are important to our friends. |  |  |  |  |
| 27 | We can learn from each other's mistakes. |  |  |  |  |
| 28 | We can share family responsibilities. |  |  |  |  |
| 29 | We firmly believe that even in difficult times, problems can be solved. |  |  |  |  |
| 30 | We tried all kinds of new ways to solve problems. |  |  |  |  |
| 31 | We can understand messages from other family members. |  |  |  |  |
| 32 | We all communicate to avoid emotional or physical harm to family members |  |  |  |  |

**The Perceived Social Support Scale (PSSS)**

The PSSS measures your subjective evaluation of support received from family, friends, and significant others. Please read each of the following 12 statements and indicate your level of agreement using the 7-point scale where 1 = Very strongly disagree, 2 = Strongly disagree, 3 = Disagree, 4 = Neutral, 5 = Agree, 6 = Strongly agree, and 7 = Very strongly agree. There are no correct or incorrect answers - simply respond according to your personal experiences. Your honest responses will help us understand your perceptions of available social support.

| **Perceived Social Support Scale (PSSS)** | | | | | | | | |
| --- | --- | --- | --- | --- | --- | --- | --- | --- |
| NO | Item | Very strongly disagree | Strongly disagree | Disagree | Neutral | Agree | Strongly agree | Very strongly agree |
| 1 | There are people (leaders, relatives, colleagues) who are there for me when I have a problem. |  |  |  |  |  |  |  |
| 2 | I can share happiness and sadness with some people (leaders, relatives, colleagues). |  |  |  |  |  |  |  |
| 3 | My family can help me in a concrete way. |  |  |  |  |  |  |  |
| 4 | I can get emotional help and support from my family when I need it. |  |  |  |  |  |  |  |
| 5 | Some people (leaders, relatives, colleagues) are a real source of comfort when I am in trouble. |  |  |  |  |  |  |  |
| 6 | My friends can really help me. |  |  |  |  |  |  |  |
| 7 | I can rely on my friends when things get tough. |  |  |  |  |  |  |  |
| 8 | I can talk to my family about my problems. |  |  |  |  |  |  |  |
| 9 | My friends can share happiness and sorrow with me. |  |  |  |  |  |  |  |
| 10 | There are people in my life (teachers, classmates, relatives) who care about my feelings. |  |  |  |  |  |  |  |
| 11 | My family is willing to help me make decisions. |  |  |  |  |  |  |  |
| 12 | I can discuss my problems with my friends. |  |  |  |  |  |  |  |

**Section 2: Demographic and Clinical Characteristics of Stroke Survivors**

**Demographic Information**

Gender

☐ Male

☐ Female

Age

☐ 18–40 years

☐ 41–60 years

☐ 61–80 years

☐ >80 years

Education Level

☐ Junior High School or below

☐ High School/Vocational School

☐ College or higher

Employment Status

☐ Employed

☐ Unemployed (voluntarily resigned)

☐ Retired

Medical Insurance Type

☐ Rural Medical Insurance

☐ Urban Medical Insurance

☐ Commercial Insurance

☐ Self-pay

Monthly Income Before Illness(RMB)

☐ <3,000 yuan

☐ 3,000–5,000 yuan

☐ >5,000 yuan

**Clinical Characteristics**

Time Since Stroke Diagnosis

☐ <1 month

☐ 1–2 months

☐ >2 months

| **Activities of Daily Living (ADL) Scale** | |
| --- | --- |
| Item | Scoring Criteria |
| 1 Eating | □ 0 = Needs full assistance |
|  | □ 5 = Needs partial help |
|  | □ 10 = Independent |
| 2 Bathing | □ 0 = Needs assistance |
|  | □ 5 = Independent |
| 3 Grooming(face washing, teeth brushing, shaving, combing) | □ 0 = Needs assistance |
|  | □ 5 = Independent |
| 4 Dressing | □ 0 = Cannot dress |
|  | □ 5 = Needs help |
|  | □ 10 = Independent |
| 5 Bowel Control | □ 0 = Incontinent |
|  | □ 5 = Occasional accidents |
|  | □ 10 = Complete control |
| 6 Bladder Control | □ 0 = Incontinent |
|  | □ 5 = Occasional accidents |
|  | □ 10 = Complete control |
| 7 Toilet Use | □ 0 = Dependent |
|  | □ 5 = Needs assistance |
|  | □ 10 = Independent |
| 8 Transfer | □ 0 = Unable |
|  | □ 5 = Major assistance needed |
|  | □ 10 = Minor assistance needed |
|  | □ 15 = Independent |
| 9 Walking | □ 0 = Immobile, Needs full assistance |
|  | □ 5 = Major assistance needed |
|  | □ 10 = Walks with assistance |
|  | □ 15 = Independent |
| 10 Stairs | □ 0 = Unable |
|  | □ 5 = Needs assistance |
|  | □ 10 = Independent |
| Total Score(Maximum 100 points ) : _____ | |
| Interpretation 评估标准: |  |
| 61-99 mild dependence |  |
| 41-60 moderate dependence |  |
| ≤40 severe dependence |  |
